# A Classification Study of Respiratory Syncytial Virus (RSV) Inhibitors by Variable Selection with Random Forest

**DOI:** 10.3390/ijms12021259

**Published:** 2011-02-21

**Authors:** Ming Hao, Yan Li, Yonghua Wang, Shuwei Zhang

**Affiliations:** 1 School of Chemical Engineering, Dalian University of Technology, Dalian, Liaoning 116012, China; E-Mails: dluthm@yeah.net (M.H.); zswei@dlut.edu.cn (S.Z.); 2 Center of Bioinformatics, Northwest A&F University, Yangling, Shaanxi 712100, China; E-Mail: yh_wang@nwsuaf.edu.cn

**Keywords:** RSV, variable selection, Mold^2^ descriptors, random forest

## Abstract

Experimental pEC_50_s for 216 selective respiratory syncytial virus (RSV) inhibitors are used to develop classification models as a potential screening tool for a large library of target compounds. Variable selection algorithm coupled with random forests (VS-RF) is used to extract the physicochemical features most relevant to the RSV inhibition. Based on the selected small set of descriptors, four other widely used approaches, *i.e.*, support vector machine (SVM), Gaussian process (GP), linear discriminant analysis (LDA) and *k* nearest neighbors (*k*NN) routines are also employed and compared with the VS-RF method in terms of several of rigorous evaluation criteria. The obtained results indicate that the VS-RF model is a powerful tool for classification of RSV inhibitors, producing the highest overall accuracy of 94.34% for the external prediction set, which significantly outperforms the other four methods with the average accuracy of 80.66%. The proposed model with excellent prediction capacity from internal to external quality should be important for screening and optimization of potential RSV inhibitors prior to chemical synthesis in drug development.

## 1. Introduction

Respiratory syncytial virus (RSV), a single-stranded RNA virus of negative genome polarity, is a member of the *Pneumovirus* genus of the *Paramyxovirus* family. RSV was first shown to occur in humans in 1957, after being recovered from two infants hospitalized with severe lower respiratory tract infections [1,2]. Today, RSV is recognized as the leading cause of virus-induced lower respiratory tract disease among infants and children [3]. Most children are infected with RSV before two years of age, re-infection is a common occurrence and morbidity due to complications is high among premature infants and those with underlying cardiopulmonary problems [4]. Moreover, RSV infections have been associated with increased prevalence of asthma in later childhood [5]. However, RSV was not recognized as a potentially serious problem in adults until the 1970s, when outbreaks of the virus occurred in long-term care facilities [6,7]. Until a safe and effective antiviral can be developed for treatment of RSV infections, prevention of the infection by use of anti-RSV antibodies appears to be the most acceptable approach. The main therapeutic agents include ribavirin [8] and RSV-IGIV [9]. However, both of them pose some disadvantages. For example, ribavirin is not a specific antiviral agent and is teratogenic, while RSV-IGIV is derived from blood, and consequently has the potential to transmit blood-borne pathogens. Thus, a search for more potent and selective inhibitors of RSV is clearly necessary. Recently, Nikitenko and co-workers have discovered a potent and selective inhibitor (RFI-641) [10]. Chapman *et al.* [11] also reported the discovery and initial development of RSV604, a novel benzodiazepine with submicromolar anti-RSV activity. In addition, with continuous efforts, Meanwell and colleagues have examined several of benzimidazole derivatives with highly potent RSV inhibition activity [12–18].

Traditionally, the biological activity of a drug candidate is obtained via costly and time consuming experiments. Thus the introduction of *in silico* methods, including the quantitative structure-activity relationship (QSAR) approaches in particular, has been explored in the drug development process for predicting the biological activity of drug candidates [19–23] prior to synthesis, thus attempting to eliminate undesirable compounds in a fast and cost-effective manner. However, to our best knowledge, there is still no report of any computational models to classify RSV inhibition activity. Therefore, it is necessary to develop a predictive model to fill this gap.

Construction of a computational model often requires two conditions. The first factor is molecular descriptors, which are used to extract the structural information that is suitable for model development. The software Mold^2^ [24] enables the rapid calculation of a large and diverse set of descriptors encoding two-dimensional chemical structure information. Comparative analysis of Mold^2^ descriptors with those calculated by Cerius^2^, Dragon or MolconnZ on several data sets has demonstrated that Mold^2^ descriptors can convey a similar amount of information as those widely-used software packages [24]. Although a freely available software, it has been proven that Mold^2^ is suitable not only for QSAR [25], but also for virtual screening large databases in drug development [24].

Secondly, the adoption of appropriate classification approaches to establish models is another central element to obtain accurate prediction. Often used classification methods include the simple but interpretable linear discriminant analysis (LDA) and partial least square (PLS) [26], and nonlinear, relatively difficult to interpret but often highly predictive methods such as artificial neural networks (ANN) [27], support vector machine (SVM), random forest (RF), Gaussian process (GP) and so forth [28–31]. All of these methods have a proven record of many successful applications in computational modeling. However, several of these methods often suffer several limitations. For example, traditional statistical method like LDA can only handle data sets where the number of descriptors (*p*) is smaller than that of the molecules (*n*), unless again a pre-selection of the descriptors is executed (e.g., by using successive projections or genetic algorithms [32,33], *etc.*). Also they are not flexible enough and do not explain nonlinear behavior [28]. SVM, a relatively new nonlinear technique employed in classification problems [34,35], is not robust to the presence of a large number of irrelevant descriptors [28]. PLS is a popular computational method that expresses a dependent variable in terms of linear combinations of the independent variables commonly known as principal components. However, PLS may not be suitable for handling multiple mechanisms of action [28], such as the nonlinear biological behaviors. Random forest, a new classification and regression tool, has been reported as combining relatively high prediction accuracy and a collection of desired features that make RF uniquely suited for modeling in cheminformatics [28] including predicting a compound’s quantitative or categorical biological activity based on a quantitative description of the compound’s molecular structure. RF has shown excellent performance even when most predictive variables are noise, and be used when the number of variables is much larger than the number of observations, and returns measures of variable importance.

It is well known that an ideal classification model should have high performance with a lower number of descriptors. Thus, in the present work, to optimize the 2D (two-dimensional) molecular descriptor subset, while simultaneously enhancing the statistical performance and efficiency of the model, the variable selection (VS) method by RF combined with backward elimination using out-of-bag (OOB) error is selected to perform a classification task for the current RSV inhibitors to investigate whether the proposed VS-RF method can construct an ideal prediction model (*i.e.*, high performance with less descriptors) for this dataset. This method was proposed originally for gene selection. The authors have proven that the novel approach can return very small sets of genes compared to the other alternative variable selection methods, while retaining predictive performance comparable to that of seven alternative state-of-art methods [36]. Although this method has been successfully applied to gene selection and microarray data [36], there is still no record of attempts to develop computational models for small molecular inhibitors. To extend the range of application, we examined the VS-RF method to classify the current dataset of RSV inhibitors. In addition, based on the performance evaluation, this method has also been compared with four other popular ones, *i.e.*, SVM, GP, LDA, and *k*NN (*k* nearest neighbors) on the basis of the selected descriptors within the same data sets.

## 2. Results and Discussion

### 2.1. Self-organizing Map

**Figure 1.**
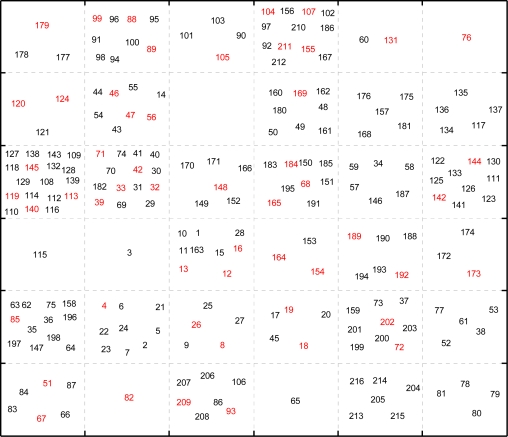
Self-organizing map (SOM) top map indicating the distribution of the training and external prediction sets. The training set is labeled in black font and the prediction set in red font. The number corresponds to the series number of the compounds of the RSV inhibitors.

As a special kind of neural network that can be used for clustering, visualization, and abstraction tasks, self-organizing map (SOM) is especially suitable for data survey due to its prominent visualization properties. In our previous work, this technology has been successfully applied to dataset split [22,31]. SOM creates a set of prototype vectors representing the dataset and carries out a topology preserving projection of the prototypes from the *d*-dimensional input space onto a low-dimensional grid [37], which is a convenient visualization space for showing the cluster structure of the data. In the present work, based on the SOM visualization of the whole data, the construction of the training and test sets was made [38]. A small Kohonen network with 6 × 6 = 36 neurons was employed, producing a map with 36 positions. All the compounds with 272 molecular descriptors were placed onto the 36 positions (neurons) of the Kohonen map. Figure 1 demonstrates the distribution of the molecules, where the number corresponds to the series number of the compounds in Table S1 (Supporting Information). The training set is labeled in black and the prediction set in red. The purpose of performing the SOM simulation on the dataset was to guarantee that the representative points of the training set are distributed evenly within the whole area of the descriptor space occupied by the dataset and the representative points of the training set are close to those of the test set, which ensures the reliability of the simulation results.

### 2.2. Selected Descriptors Using VS-RF

A VS-RF strategy has been developed successfully, with the final number of descriptors being reduced to six from the original 272 for the further study. Since it is recommended that the number of compounds in the training set should be at least five-times larger than that of the selected independent variables [39], the model developed by VS-RF obviously maintains the recommended ratio. Table 1 lists the selected descriptors together with their definitions and their values are listed in Table S2 (Supporting Information).

### 2.3. Performance of Different Statistical Methods

Based on the selected descriptors, five different statistical methods (VS-RF, SVM, GP, LDA, *k*NN) were performed to compare their performance, and the detailed statistics are summarized in Table 2. The results predicted by these methods are presented in Table S3 (Supporting Information).

VS-RF: Random forest effectively has only one tuning parameter, *m*_try._ In the present work, the *m*_try_ value was tried from 1 to 6 and the optimal value determined by 10-fold cross-validation accuracy (Q_cv_ = 0.816). Ultimately, optimal RF results are obtained based on the *m*_try_ = 4 and 500 trees in the forest. The efficiency and robustness of the derived models are further evaluated by using the external prediction set. As shown in Table 2, for the external prediction set, the prediction accuracies of VS-RF are 100% for high active RSV inhibitors and 88.46% for low active ones, with a total accuracy (Q) of 94.34%. The values of MCC and F are 0.89 and 0.96, respectively.

SVM: Similar to other multivariate statistical models, the performance of SVM depends on the combination of several parameters including the capacity parameter *C*, the kernel type K and its corresponding indices. *C* is a regularization parameter which controls the tradeoff between maximizing the margin and minimizing the training error. In this work, the grid search technology was employed to obtain the optimum parameters (*C* and sigma) using the R package caret [40] on the basis of 10-fold cross validation. Here, the function sigest in the kernlab package [41] was used to provide a good estimate of the sigma parameter, so that only the *C* parameter was tuned. The final values used in the model are *C* = 10 and sigma = 0.284 with the highest 10-fold cross-validation accuracy (0.791). Using the determined optimal parameters, the SVM obtains statistical results of 85.19%, 80.77% and 83.02% for the sensitivity, specificity and Q of the test set, respectively. The MCC and F values are 0.66 and 0.84, respectively.

GP: The Gaussian process method, based on clearly defined statistical principles and easily programmed [42], was also adopted to classify the RSV-related compounds. The optimal inverse kernel width for the Radial Basis kernel function (sigma) was finally fixed to 0.284 based on sigest function including the R package kernlab. Based on the 10-cross-validation, the final Q_cv_ of GP we derived is 0.78. As for the RF model, the GP model also presents 100% sensitivity, however, a low specificity of 76.92% for the test set. In addition, the values of Q, MCC and F are 88.68%, 0.79 and 0.9, respectively.

LDA: a widely used classification technology, LDA, was also performed to classify the current dataset based on the selected six descriptors. As shown in Table 2, no statistically satisfactory LDA-based model could be obtained, with the optimal one only depicting sensitivity of 74.07%, specificity of 80.77%, and overall accuracy of 77.36% for the test set. The value of Q_cv_ was just 0.675.

*k*NN: After 10-fold cross-validation, an optimal *k* = 17 was determined on the basis of the highest accuracy (Q_cv_ = 0.729). As seen from Table 2, the sensitivity and specificity for the prediction set are 81.48% and 65.38%, respectively. And the overall prediction accuracy for the test set is 73.58%. The values of MCC and F are 0.48 and 0.76. It is obvious that *k*NN, of the five statistical methods, is uniformly less able to predict than the others.

### 2.4. Comparison of Different Approaches

From the above discussion, it can be concluded that the developed VS-RF model performed comparably with SVM and GP, demonstrated by the Q_cv_(%) of VS-RF, SVM and GP of 81.6%, 79.1% and 78%, respectively, in terms of cross-validation. These models outperform those of the LDA and *k*NN, whose Q_cv_(%) are 67.5% and 72.9%, respectively. High cross-validation accuracy is necessary, but not sufficient for a model with high predictive ability [43], thus an external validation is a better way to estimate the performance of the models. Therefore, a further investigation of Q(%) in the external prediction set was performed, where the VS-RF model increases about 11.32% and 5.66% compared to the SVM and GP models, respectively. It should be noted that although GP shares the same prediction ability for high active compounds, for low active inhibitors the prediction accuracy decreases by 11.54% compared with VS-RF. From this point of view, one can consider that the VS-RF model is more favorable than others for the RSV inhibitors.

**Figure 2.**
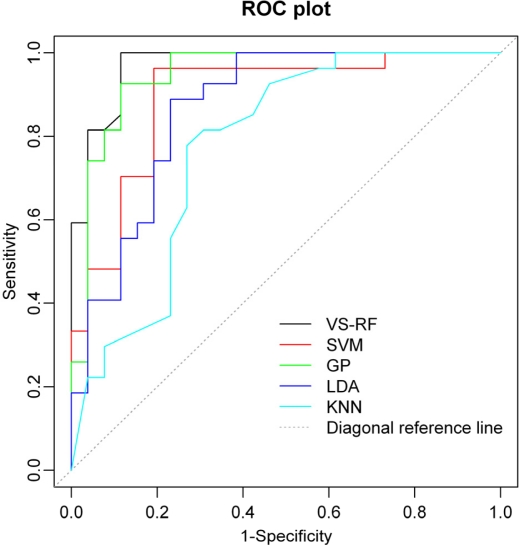
The ROC (receiver operating characteristic) curves of VS-RF, SVM, GP, LDA and *k*NN for the prediction set.

In addition, when comparing the other four models, it is observed that the LDA model is comparable to that of *k*NN, both of them presenting less overall accurate (Q) (77.36% for LDA and 73.58% for *k*NN) in the test set than the other models. The reason for LDA’s failure may be due to the existence of some nonlinear relationship between the molecular structures and the corresponding activity. For *k*NN, a possible reason for the low accuracy is that the method—based on the Euclidean distance—may not be the most effective approach for every problem just like the present one. Furthermore, for SVM and GP, their internal prediction ability is comparable, while the performance of GP is slightly better than SVM in terms of the external prediction. The area under the ROC (receiver operating characteristic) curve (AUC) [44,45] is also considered as an important criterion for measuring the performance of the model. An AUC value of 1 indicates a theoretically perfect performance, while a value of 0.5 denotes no prediction ability. Clearly, the closer the AUC value is to 1, the better the model performance is. Figure 2 gives the ROC curves of VS-RF, SVM, GP, LDA and *k*NN for the prediction set. The computed AUC values for the five statistical methods are 0.96, 0.89, 0.94, 0.86 and 0.78, respectively, also proving the good prediction ability and reliability of the VS-RF model. Thus, our further analysis is only restricted to the VS-RF model for prediction of RSV inhibition.

It should be noted that RF, as a new classification and regression tool, can well solve the small *n* and large *p* (*n* < *p*, that is the number of samples is smaller than that of descriptors) problems [28] even without variable selection. Keeping this in mind, in order to estimate the effect of VS-RF, we have compared both the statistical performance with and without variable selection. As shown in Table 3, for the training set, the statistical performance obtained with or without variable selection makes no difference, while the time cost of RF is approximately 20-times more than that of VS-RF. It must be pointed out that for the RF model without variable selection, the optimal *m*_try_ is obtained using grid search technology including the R package caret, and the search length is set to 10. For the test set, one can see that the statistics of VS-RF outperform RF. The VS-RF presents a sensitivity of 100%, while RF gives that of 92.59%, that is to say there are two high active compounds misclassified to low active ones by RF. According to above analysis, one can conclude that the VS-RF model depicts not only high computation efficiency but also enhances prediction ability. Therefore, for the RSV inhibitor classification, the VS-RF model gives very high statistical results with total accuracies of 100% and 94.34%, for the training and test set, respectively. In the final VS-RF model, three compounds (No. 68, 120 and 124) are misclassified (Tables S1 and S3; Supporting Information). The reason for misclassification of compound 68 is unclear, since by comparison with compound 39, the former introduces a polar substituent CH_2_COOH instead of Et, however, the activity decreases sharply suggesting the atomic polarizabilities may play a role in the RSV inhibition. Compounds 120 and 124 are misclassified as high active molecules by the VS-RF model. By investigation of the correctly classified compounds, *i.e.*, 115, 116, 118, 119, 123 and 125∼132 in Tables S1 and S3 (Supporting Information), it is revealed that all of them possess a linear R1 group at position 5. However, compounds 120 and 124 have a ring-based substituent at the same location, which we suppose may be the reason for the misclassification.

### 2.5. Interpretation of the Selected Descriptors

By using feature selection, the most appropriate sets of molecular descriptors for predicting the RSV low and high active inhibitors are extracted from the VS-RF models, some of which probably provide new insights into the physicochemical characteristics of RSV inhibition by specific classes of compounds. D299, one of the topological descriptors, is a molecular branching index that is calculated from the algebraic formulas derived by Lovasz and Pelikan for special types of trees such as path or star and for particular eigenvalues [46]. The highest molecular branching corresponds to the most branched graphs. This is in agreement with the previous result that the topology of the side chain is important to modulate physical properties [12]. D347 stands for molecular topological path index of order 07. The path counts are molecular descriptors obtained from an H-depleted molecular graph and are vertex invariants encoding that molecular environment, defined as the number of path lengths *m* starting from the *i*th vertex to any other vertex in the graph. A path (or self-avoiding walk) is a walk without any repeated vertices [47]. The path length is the number of edges associated with the path, and this value is increased with the ring size, ring numbers, and the ramification number [48]. Of the selected six descriptors, D503 and D513 belong to 2D autocorrelation classes, which represent the topological structure of the compounds but are more complex in nature than the classical topological descriptors. Computation of these descriptors involves the summations of different autocorrelation functions corresponding to different structural lags and leads to different autocorrelation vectors corresponding to the lengths of sub-structural fragments. Hence, it can distinguish the details of important sub-structural differences. In the previous work, the 2D autocorrelation descriptors have been proven advantageous for establishing a QSAR model [49–53]. For the present work, the Moran’s index *I* [53,54] is employed for the classification of RSV inhibitors:

(1) $$I=\frac{n}{2L}\frac{\sum_{\text{ij}}\delta_{\text{ij}}(p_{\text{ki}}-{\bar{p}}_{k})(p_{\text{kj}}-{\bar{p}}_{k})}{\sum_{i}\left(p_{\text{ki}}-{\bar{p}}_{k}\right)}$$

where *n* is the total number of data points; *p_ki_* and *p_kj_* are the values of physicochemical properties (*i.e.*, atomic van der Waals volumes, and atomic polarizabilities in the present work) *k* of atoms *i* and *j*, respectively; *p̄_k_* is the average value of property *k*; and *δ_ij_* is a Dirac-delta function defined as

(2) $$\delta_{\text{ij}}=\{\begin{array}{ll} 1 & \text{if}\;d_{\text{ij}}=1\\ 0 & \text{if}\;d_{\text{ij}}\neq 1 \end{array}$$

where *d*_ij_ is the topological distance of spatial lag between atoms *i* and *j*.

The 2D autocorrelation descriptors can be obtained by summing up the products of certain properties of the two atoms located at a given topological distance or spatial lag. The most important factor in interpreting them in the model is the topological distance, once weighted equally. In point of this fact, the best model selected an optimum descriptor combination, which includes van der Waals volumes and atomic polarizabilities as the most relevant key features (Table 1). This result illustrates that a certain distribution of these properties is necessary to distinguish the RSV inhibitors.

The last selected two descriptors (D513 and D528) belong to topological charge indices. D513, molecular topological order-3 charge index (GGI3) represents the three eigenvalues of the corrected adjacency matrix of a molecule. D528, the mean molecular topological order-8 charge index (JGI8), is a kind of Galvez topological charge index which evaluates the charge transfers between pairs of atoms and the global charge transfers in the molecule [55]. Galvez charge indices GGIK and JGIK are computed as follows:

(3) $$\text{GGIK}=\sum_{i=1,\;j=i+1}^{i=N-1,\;j=N}\left|{\text{CT}}_{\text{ij}}\right|\delta (k,D_{\text{ij}})$$

(4) $$\text{JGIK}=\frac{\text{GGIK}}{N-1}$$

where *N* is the number of vertices (atoms different to hydrogen) in the molecular graph, and *k* the length of each path. *CT_ij_* = *m_ij_* – *m_ji_ m* stands for the elements of M matrix, M = *A* × D^*^ where *A* is the adjacency (*N* × *N*) matrix of the molecular graph and D^*^ is the inverse square distance matrix in which their diagonal entries are assigned as 0, and *δ* is Kronecker’s delta. Thus, JGIK represents the average of the *CT_ij_* terms with *D_ij_* = *k*, being *D_ij_* the entries of the topological distance matrix (*D*). In the Charge Indices terms, the presence of heteroatoms is taken into account by introducing their electronegativity values in the corresponding entry of the main diagonal of the adjacency matrix. These indices represent a strictly topological quantity plausibly correlating with the charge distribution inside the molecule. This distribution is an important property, which conditions the behavior of many physiochemical and biological properties. This index describes topological characteristics of the molecules.

From the aforementioned discussion, it can be seen that the activity of these RSV inhibitors is mainly influenced by several factors including the molecular branching index and atomic polarizabilities. These results are to some extent in agreement with the corresponding related experimental conclusions [12,13,18]. For example, Yu *et al.* reported that the topology of the side chain of RSV inhibitors is important, while we also find that the corresponding descriptors (D299 and D347) play a part in RSV inhibition. The studies on a series of benzotriazole derivatives as RSV inhibitors [13] revealed a broad tolerance for substituent size and functionality, our selected 2D autocorrelation descriptors also disclose such information. In reference [12], the authors reported that the polar functionality provides considerable latitude to modulate both the pharmaceutical and pharmacokinetic properties, which is found also to be of considerable importance in the quest for orally effective RSV inhibitors. In addition, reference [18] illustrated polarity in the oxime substituent in a series of compounds with potent antiviral activity in cell culture that combined good metabolic stability *in vitro* with high cell membrane permeability, and the descriptor D503 also depicts the role that atomic polarizabilities plays in RSV inhibition.

As expected, besides the robust, sparse and predictive features, an ideal classification model would still be interpretable. In many cases, gaining an intuitive interpretation of important features from the two-dimensional QSAR is not always simple. For the present work, it should be pointed out that our explanations for the current descriptors are just broad due to nonlinear model types and abstract descriptors. However, in terms of developing a highly predictive classification model, the proposed VS-RF model in this work could allow this task.

## 3. Material and Experimental Methods

### 3.1. Data Sets

A large, diverse dataset of 216 RSV inhibitors collected from the literature [12–18] published by the same research group with converted molar pEC_50_ (−logEC_50_) values ranging from less than 3.563 to 8.699 mole were used as the dataset in the present study. These EC_50_ values were the results of two experiments performed on consecutive weeks with the data from individual experiments shown in parentheses. Based on the inhibitory activity, the dataset is split into two classes, *i.e.*, 107 low active compounds with pEC_50_ < 6.5 and 109 high active ones with pEC50 > 7.5. Table 4 depicts several representative compounds together with their classification labels. All information of the dataset with their diverse scaffolds of structures is provided in Table S1 (Supporting Information).

### 3.2. Descriptors Calculation and Pre-processing

In the present work, the two dimensional structures of all RSV inhibitors were built with the ISIS/Draw 2.3 program [56], and converted to SDF format by Open Babel software package (http://openbabel.sourceforge.net/). The final structures were transferred into Mold^2^ [24], a free program available to public to calculate molecular descriptors. The Mold^2^ software package can calculate 777 molecular descriptors solely from 2D chemical structures, and the models generated using Mold^2^ descriptors were reported comparable to those generated using descriptors from the compared commercial software packages [24]. In our work, all original 777 Mold^2^ molecular descriptors were calculated, and then underwent a pre-processing process (also called unsupervised selection of descriptors) as follows: (1) descriptors containing larger than 85% zero values were removed; (2) zero- and near zero- variance predictors were removed because such descriptors may cause the model to crash or the fit to be unstable; and (3) one of the two descriptors that have the absolute correlations above 0.95 was omitted. After these steps, the number of original descriptors was reduced to 272 for further research.

### 3.3. Split of the Training and Test Sets

Rational division of an experimental SAR (structure-activity relationship) dataset into the respective training and test sets for model development and validation is very important. The methods often used include random sampling (RS), Kennard-Stone (KS), K-mean clustering, and self-organizing map, *etc.* The basic rule should be that the points of the training set are distributed evenly within the whole area covered by the dataset, and that the condition of closeness of the test set points to those of the training set is satisfied [57].

For the independent prediction set, we performed our selection on the basis of their distribution in the chemical space, which is defined by Kohonen neural network [58]. The Kohonen neural network of dimension 6 × 6 was applied, which enables one to map objects into 36 positions. Similar objects were mapped into the same position (*x*, *y* coordinates in a Kohonen map). Only one part of a representative object from each position in the Kohonen map was chosen for the training set, respecting the original proportion among the different classes and the predefined 3:1 ratio between the training and the test objects. The rest were put into the test set. The self-organizing map simulations were carried out using internally developed C-language program. The training set was used for the development of the classification models, and the independent prediction set was used for the assessment of the system. The training and independent test sets contain 163 (81 low active and 82 high active) and 53 (26 low active and 27 high active) compounds, respectively, with approximately one-fourth of the respective groups assigned in the independent prediction set.

### 3.4. Statistical Methods

VS-RF: Random forest model was constructed according to the described original RF algorithm [59]. RF is an ensemble of single decision trees, whose assembly produces a corresponding number of outputs and the outputs of all trees are aggregated to obtain one final prediction. The training algorithm of the RF for classification can be briefly summarized as follows: (1) Draw *N* bootstrap samples from the original training set. (2) Construct an unpruned tree *T_p_* (*p* = 1, …, *N*) with each training set *B_p_*. At each node, rather than choosing the best split among all predictors, randomly sample *m*_try_ of the predictors and then choose the best split from among those variables. The tree is grown to maximum size and not pruned back. (3) Predict the *N* trees by majority vote for classification. RF algorithm is the same as Bagging when *m*_try_ = *p* and the tree growing algorithm used in RF is CART (classification and regression tree). The RF algorithm can be efficient especially when the number of descriptors (*p*) is very large. This is because RF only tests the *m*_try_ of the descriptors rather than the *p*, where the default *m*_try_ is the square root of the number of descriptors for classification. Thus, *m*_try_ is very small, so that the search is very fast.

RF possesses its own reliable statistical characteristics based on OOB set prediction, which could be used for validation and model selection with no cross-validation performed. It was shown that the prediction accuracy of an OOB set and a 5-fold cross validation procedure was nearly the same [28]. Although RF performs relatively well “off the shelf” without expending much effort on parameter tuning or variable selection [28], it is also important for carrying out some tentative investigations on the changes of *m*_try_ or descriptor selection to optimize the performance of RF. In the current study, the optimal *m*_try_ was determined when the prediction accuracy reached the highest based on the 10-fold cross-validation.

Random forest, as a new classification and regression tool, has not been frequently applied in QSAR, QSPR (quantitative structure-property relationship) [25,28,60,61]. Thus it should be of value to investigate whether the RF can be applied to obtain better statistical performance for the current dataset of RSV inhibitors. Here, only a brief introduction about RF is presented, since more details can be found in corresponding literatures [28,59]. In the present work, the RF algorithm was employed using the R package randomForest [62].

As expected, an ideal classification model should possess high prediction ability with a small set of descriptors. Thus, variable selection with random forest was used to implement this task. Here, we simply introduce the VS-RF. To select optimal descriptors, random forests were iteratively fitted, at each iteration building a new forest after discarding those descriptors with the smallest variable importance; the selected set of descriptor is the one that yields the smallest OOB error rate. In this algorithm, all forests result from eliminating, iteratively, a fraction, *fraction.dropped*, of the descriptors (the least important ones) used in the previous iteration. By default, *fraction.dropped* = 0.2, which allows for relatively fast operation, coherent with the idea of an “aggressive variable selection” approach, and increases the resolution as the number of descriptors considered becomes smaller. After fitting all forests, the OOB error rates from all the fitted random forests were examined. And the solution with the smallest number of descriptors whose error rate is within *μ* standard errors of the minimum error rate of all forests is chosen. Setting *μ* = 0 is the same as selecting the set of descriptors that leads to the smallest error rate. Setting *μ* = 1 is similar to the common “1 s.e. rule”, used in the classification trees [36]. In our work, the *μ* = 1 was adopted, since this strategy can lead to solutions with fewer descriptors than selecting the solution with the smallest error rate, while achieving an error rate that is not different, within sampling error, from the “best solution”. More details on the VS-RF can be found in literature [36]. The variable selection from random forest was performed using the R package varSelRF [63]. All parameters were adopted by default.

SVM: Support vector machines are a relatively new type of learning algorithm originally introduced by Vapnik and co-workers [64]. Due to its many attractive features and promising empirical performances, SVM is gaining increasing popularity in many fields [65,66], and thus was also performed in the present work. Since there have been a number of excellent introductions into SVM [35,64,67], only a briefly description of the main idea of SVM classification is presented here.

For the classification task, briefly, this involves the optimization of Lagrangian multipliers *α_i_* with constraints 0 ≤ *α_i_* ≤ *C* and ∑*α_i_ y_i_* = 0 to yield a decision function as follows:

(5) $$f(x)=\text{sign}(\sum_{i=1}^{l}y_{i}\alpha_{i}K(x,x_{i})+b)$$

where *y_i_* are input class labels that take a value of −1 or 1, *x_i_* are a set of descriptors, and *K* (*x*, *x_i_*) is a kernel function, whose value is equal to the inner product of two vectors *x* and *x_i_* in the feature space Φ(*x*) and Φ(*x_i_*), *i.e.*, *K*(*x*, *x_i_*) = Φ(*x*) × Φ(*x_i_*). The elegance of using a kernel function lies in the fact that one can deal with feature spaces of arbitrary dimensionality without having to compute the Φ(*x*) explicitly. Any function that satisfies Mercer’s condition can be used as the kernel function. The sign function *sign*(*μ*) returns 1 when *μ* > 0, and −1 when *μ* ≤ 0. In support vector classification, the Gaussian kernel *K*(*μ*, *υ*) = exp(−|*μ* – *υ*|^2^ / *δ*^2^) was used. And the R package kernlab was used to develop the SVM classification model.

GP: Preliminarily used in QSAR field, the Gaussian process (GP) was also introduced in the present study to classify the RSV inhibitors. Pioneering work was made by Burden [42] who demonstrated GP applications in QSAR modeling of data sets of compounds active at the benzodiazepine and muscarinic receptors, *etc.* In addition, the authors of these references [68–71] have also reported the successful use of GP in statistical predictions of a series of pharmacokinetic properties. Recently, GP was also reported to be applied both in an automatic QSAR modeling of ADME (absorption, distribution, metabolism, excretion) properties [72], and the multivariate spectroscopic calibration [73]. All these works confirmed the possibility of GP as a promising machine learning tool, to be used in QSAR studies. In view of this, the present study is dedicated to introducing GP in classification modeling of RSV inhibitors.

A Gaussian process is defined simply as a collection of random variables which have a joint Gaussian distribution. It is completely characterized by its mean and covariance function. In the GP, the kernel function used in training and prediction contains (1) Radial Basis kernel function “Gaussian”; (2) Polynomial kernel function; (3) Linear kernel function; (4) Hyperbolic tangent kernel function; (5) Laplacian kernel function; (6) Bessel kernel function; (7) ANOVA RBF kernel function; and (8) Spline kernel. In the present work, the popular Radial Basis kernel function was chosen, with the kernel parameters determined by sigest function implemented in the R package kernlab.

LDA: LDA is a pattern recognition method providing a classification model based on the combination of variables that best predicts the category or group to which a given compounds belongs. The basic theory of LDA is to classify the dependents by dividing an *n*-dimensional descriptor space into two regions that are separated by a hyperplane defined by a linear discriminant function. In this study, the independent variables were the calculated molecular descriptors, and the discrimination property was EC_50_ (represented by either high active or low active). Statistical analyses were performed using the R package MASS [74].

*k*NN: *k*NN measures the Euclidean distance between a to-be-classified vector *x* and each individual vector *x*_i_ in the training set [75]. A total of *k* number of vectors nearest to the vector *x* are used to determine its class, *f*(*x*):

(6) $$\hat{f}(x)\leftarrow \text{arg}\;{\text{max}}_{v\in V}\;\sum_{i=1}^{k}\delta [v,\;f(x_{i})]$$

where *δ*(*a*,*b*) = 1 if *a* = *b* and *δ*(*a*,*b*) = 0 if *a* ≠ *b*, argmax is the maximum of the function, *V* is a finite set of vectors {*v*_1_,...*v_s_*}, and *f̂* (*x*) is an estimate of *f*(*x*). Here, estimate refers to the class of the majority of the *k*NNs. Here, the *k*NN computation was performed by R package caret [40].

### 3.5. Evaluation of the Statistical Performance

As in the case of all discriminative methods [22,31], the performance of statistical learning methods can be measured by a series of parameters including the quantity of true positives (TP), true negatives (TN), false positives (FP), false negatives (FN), sensitivity (SE) (also called recall), SE=TP/(TP + FN), which is the prediction accuracy for the high active compounds in this work, and specificity (SP), SP = TN/(TN + FP), which is the prediction accuracy for the low active inhibitors, Precision = TP/(TP + FP), which is the positive predictive value. The overall prediction accuracy (*Q*), Matthews correlation coefficient (*MCC*) and *F*-measure, a function of recall and precision which indicated the accuracy of real and estimated class, respectively, are also used to measure the prediction accuracies and can be given as follows:

(7) $$Q=\frac{\text{TP}+\text{TN}}{\text{TP}+\text{TN}+\text{FP}+\text{FN}}$$

(8) $$\text{MCC}=\frac{\text{TP}\times \text{TN}-\text{FN}\times \text{FP}}{\sqrt{\left(\text{TP}+\text{FN})(\text{TP}+\text{FP})(\text{TN}+\text{FN})(\text{TN}+\text{FP}\right)}}$$

(9) $$F-\text{measue}=\frac{2\times \text{recall}\times \text{precision}}{\text{recall}+\text{precision}}$$

## 4. Conclusions

In the present work, based on the up-to-date largest dataset (to our best knowledge) of 216 structurally diverse RSV inhibitors, a VS-RF classification model with good predictive performance (the overall Q = 94.34% for the prediction set) has been built.

By explanation of the selected descriptors, we conclude that the topological structure and electronic factors play a central role in the RSV inhibition. Moreover, a comparison with four other statistical methods, *i.e.*, SVM, GP, LDA and *k*NN, demonstates that the VS-RF model presents better statistics both for the training and test sets. Through a comparison of RF statistical performance with and without variable selection based on these RSV inhibitors, the proposed VS-RF method not only improves the prediction ability but also enhances computational efficiency. Therefore, we hope that this method and the derived model will be of help for predictive tasks to screen new and potent RSV inhibitors in early drug development.

## 





## Figures and Tables

**Table 1.** The selected 6 Mold^2^ descriptors using variable selection algorithm coupled with random forests (VS-RF) and their definition.

**Descriptor**	**Definition**	**Class**
D299	The largest eigenvalue	Eigenvalue-based indices
D347	Molecular topological path index of order 07	Walk and path counts
D490	Moran topological structure autocorrelation length-4 weighted by atomic van der Waals volumes	2D autocorrelation
D503	Moran topological structure autocorrelation length-1 weighted by atomic polarizabilities	2D autocorrelation
D513	Molecular topological order-3 charge index	Topological charge indices
D528	Mean molecular topological order-8 charge index	Topological charge indices

**Table 2.** The prediction performance of high and low active compounds as respiratory syncytial virus (RSV) inhibitors from VS-RF, SVM, GP, LDA and *k*NN statistical methods for the external prediction set and the 10-fold cross-validation^a^.

**Model**	**High active inhibitors**			**Low active inhibitors**			**Q (%)**	**MCC**	**F**	**Q_cv_ (%)**
	**TP**	**FN**	**SE (%)**	**TN**	**FP**	**SP (%)**				
VS-RF	27	0	100	23	3	88.46	94.34	0.89	0.96	81.6
SVM	23	4	85.19	21	5	80.77	83.02	0.66	0.84	79.1
GP	27	0	100	20	6	76.92	88.68	0.79	0.9	78
LDA	20	7	74.07	21	5	80.77	77.36	0.55	0.77	67.5
*k*NN	22	5	81.48	17	9	65.38	73.58	0.48	0.76	72.9

a, VS-RF, *m*_try_ = 4; SVM, *C* = 10, sigma = 0.284; GP, sigma = 0.284; *k*NN, *k* = 17; TP, true positives; FN, false negatives; SE, sensitivity; TN, true negatives; FP, false positives; SP, specificity; Q, the overall prediction accuracy; MCC, Matthews correlation coefficient; F, F-measure; Q_cv_, the prediction accuracy from 10-fold cross-validation for the training set.

**Table 3.** Comparison of random forest (RF) statistical performance with and without variable selection based on the respiratory syncytial virus (RSV) inhibitor dataset a.

		**High active inhibitors**			**Low active inhibitors**					
**Model**		**TP**	**FN**	**SE(%)**	**TN**	**FP**	**SP(%)**	**Q(%)**	**Q_cv_**	**Time cost (s)**
Training set	RF	82	0	100	81	0	100	100	0.816	171.42
	VS-RF	82	0	100	81	0	100	100	0.816	8.06
Test set	RF	25	2	92.59	23	3	88.46	90.57	-	-
	VS-RF	27	0	100	23	3	88.46	94.34	-	-

a, for RF, *m*_try_ = 62; for VS-RF, *m*_try_ = 4; TP, true positives; FN, false negatives; SE, sensitivity; TN, true negatives; FP, false positives; SP, specificity; Q, the overall prediction accuracy; MCC, Matthews correlation coefficient; F, F-measure; Q_cv_, the prediction accuracy from 10-fold cross-validation for the training set.

**Table 4.** Representative compounds with their chemical names, activities and classes used in the dataset.

**No.**	**Structure**	**pEC_50_**	**Class^b^**	**Ref.^a^**
1	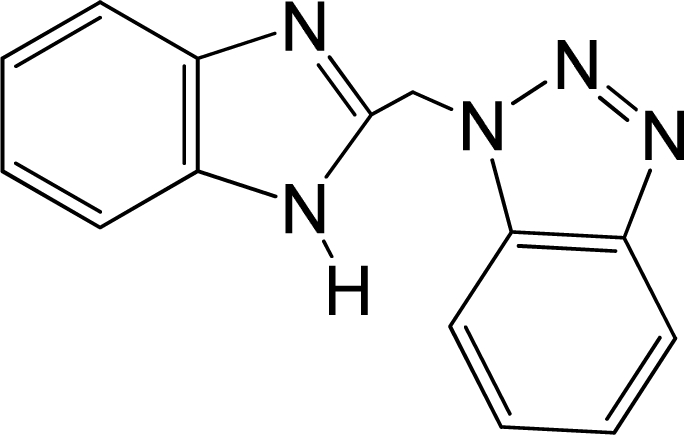	4.507	L	12
2	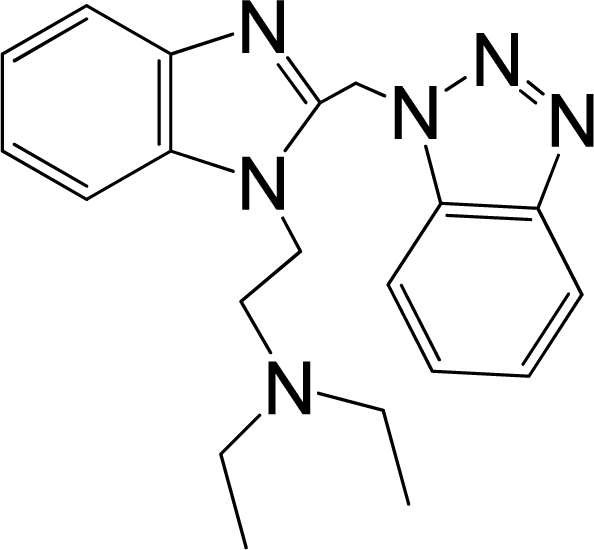	6.328	L	12
3	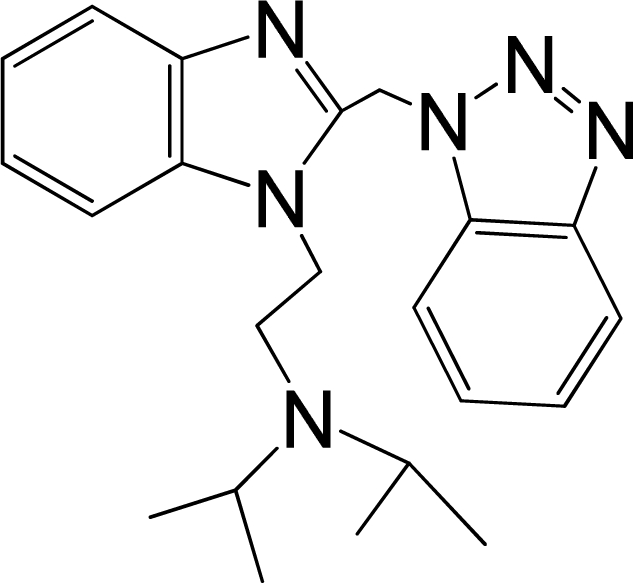	5.174	L	12
4^*^	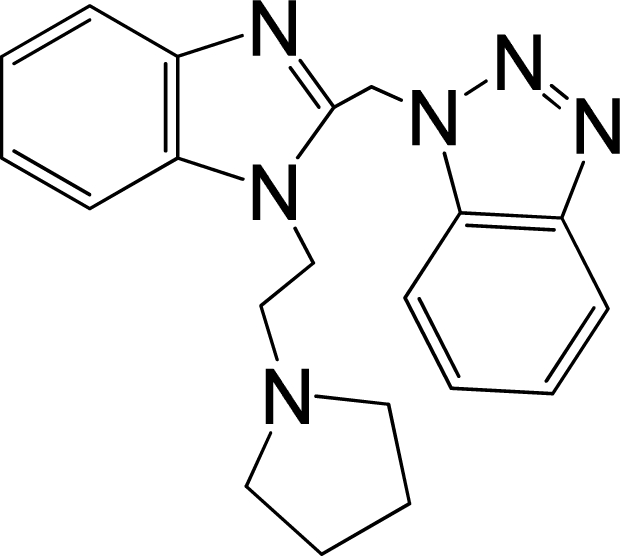	6.222	L	12
5	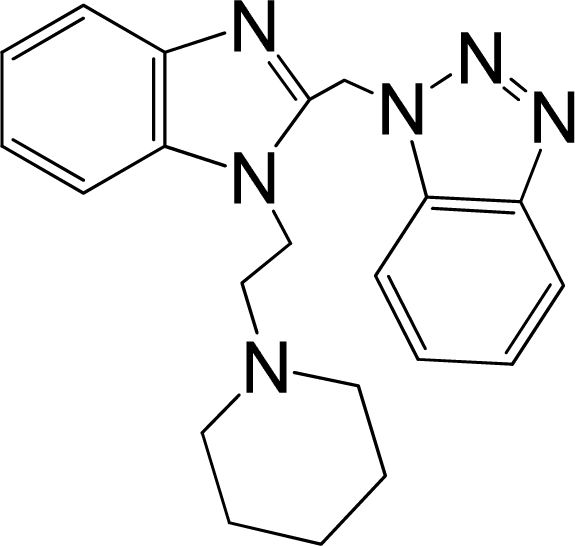	5.959	L	12
7	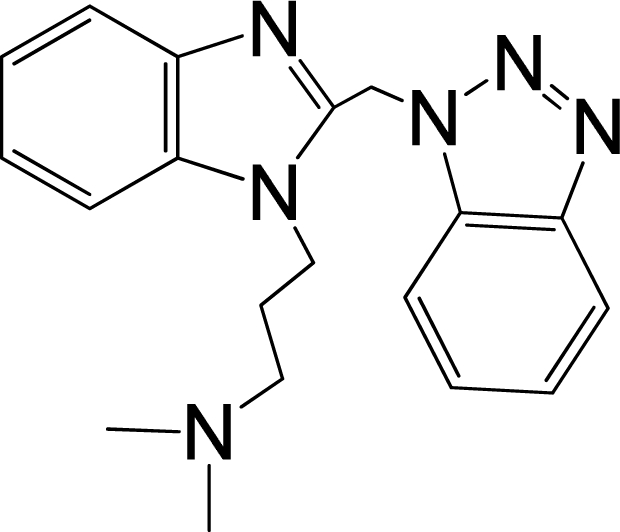	5.959	L	12
8^*^	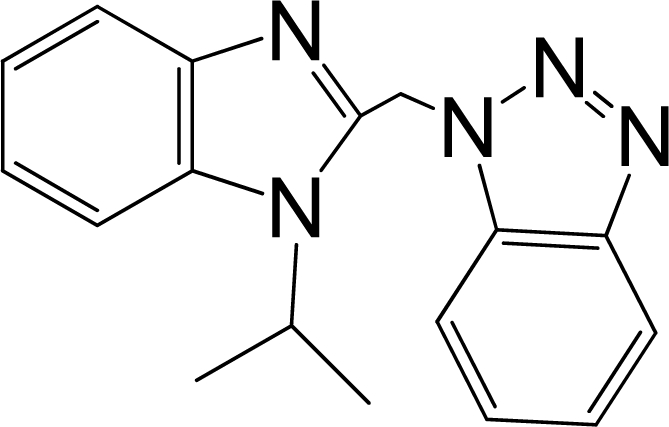	4.81	L	12
9	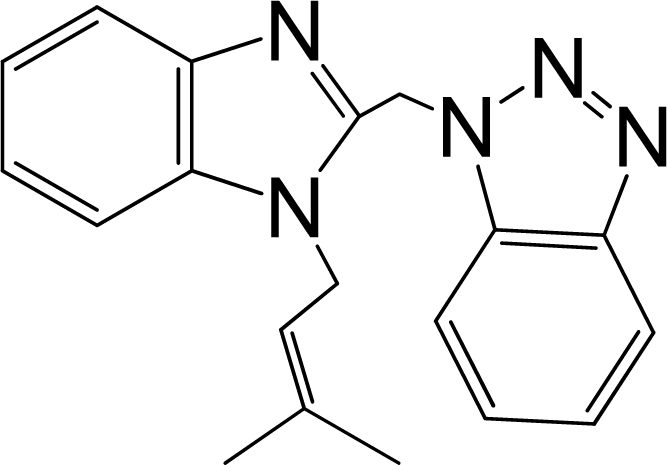	5.481	L	12
10	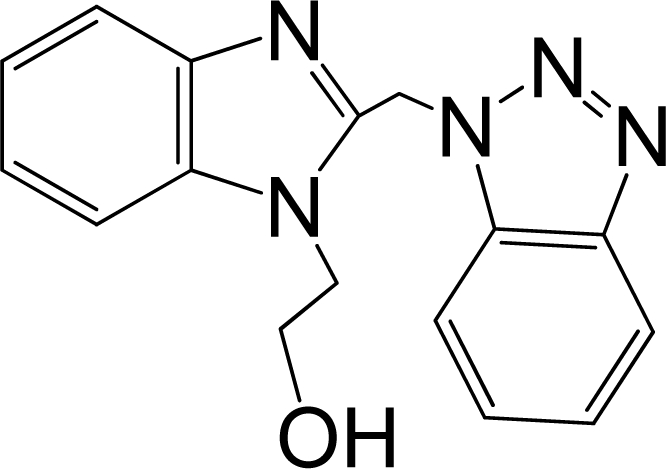	5.114	L	12
11	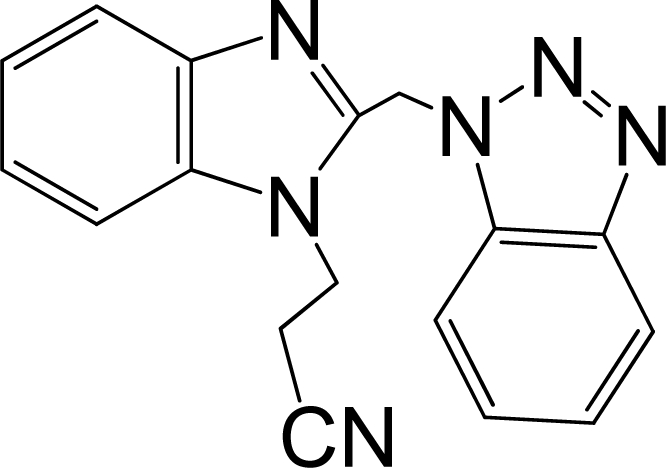	5.570	L	12
12^*^	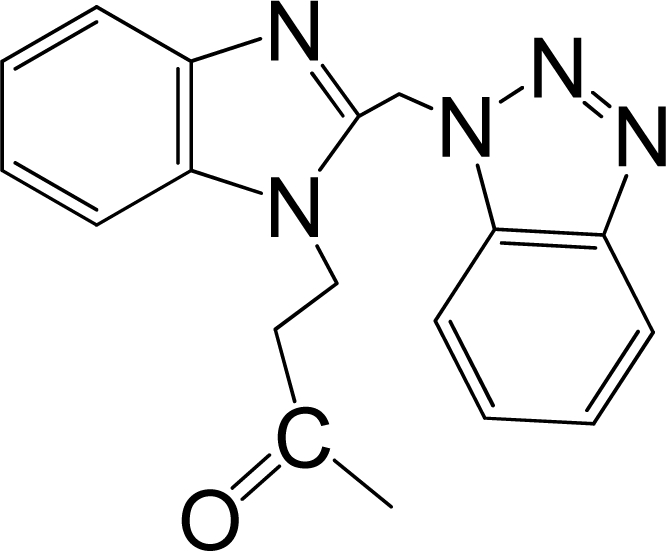	6.284	L	12
29	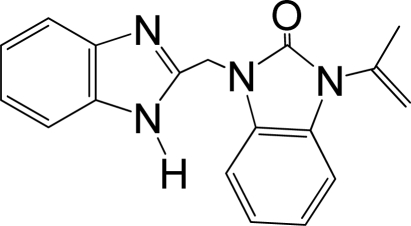	6.125	L	13
30	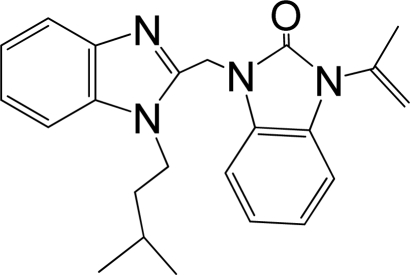	8.398	H	13
31	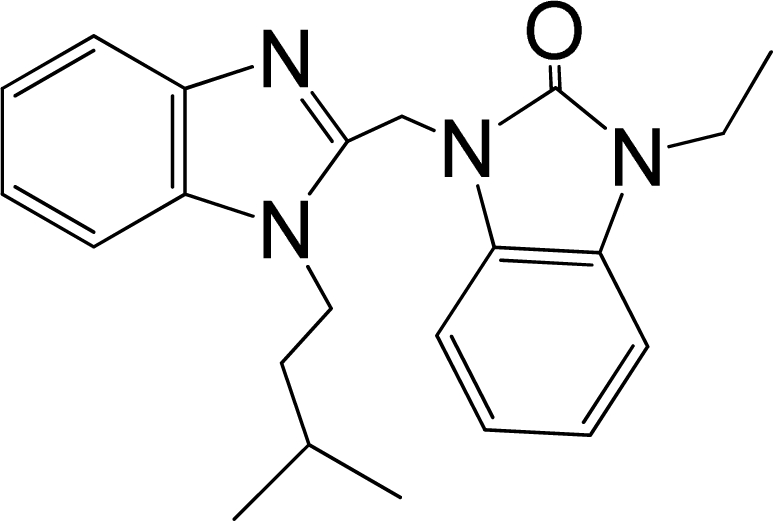	7.959	H	13
32^*^	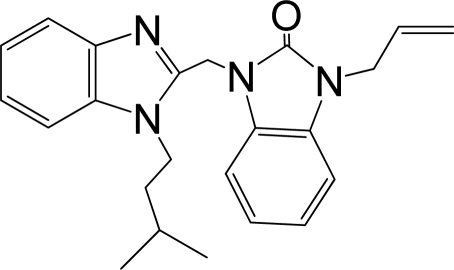	7.796	H	13
34	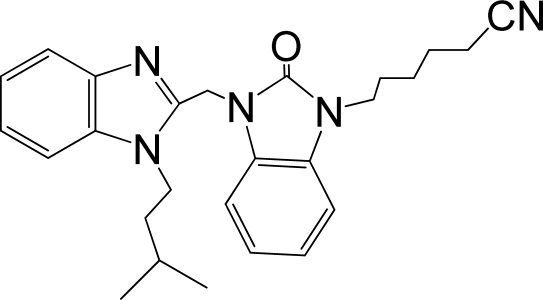	7.602	H	13
35	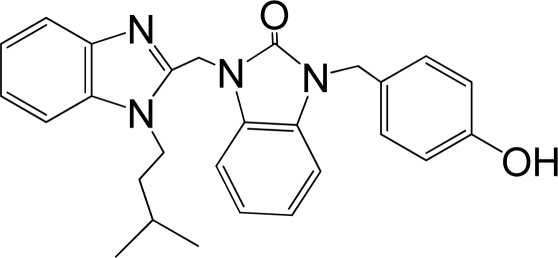	7.745	H	13
36	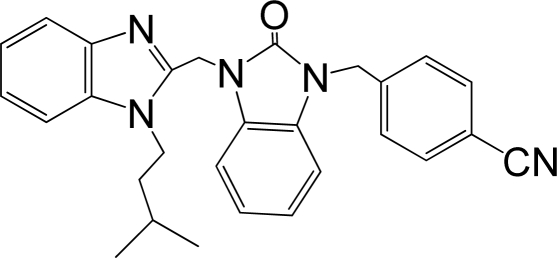	7.921	H	13
37	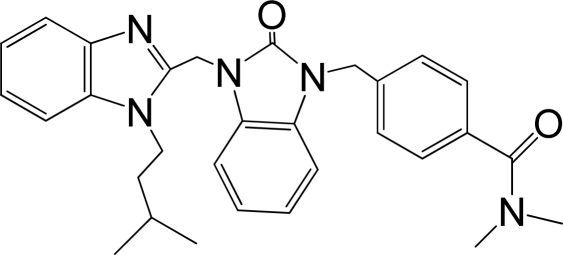	7.678	H	13
38	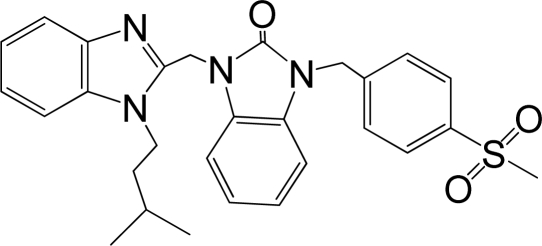	8.046	H	13
39^*^	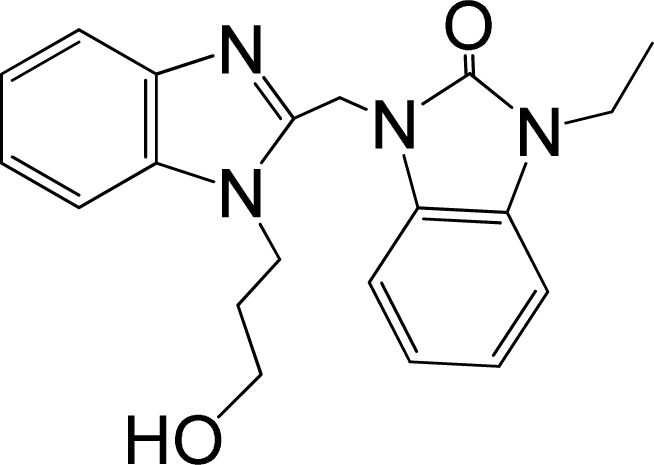	8.000	H	13
41	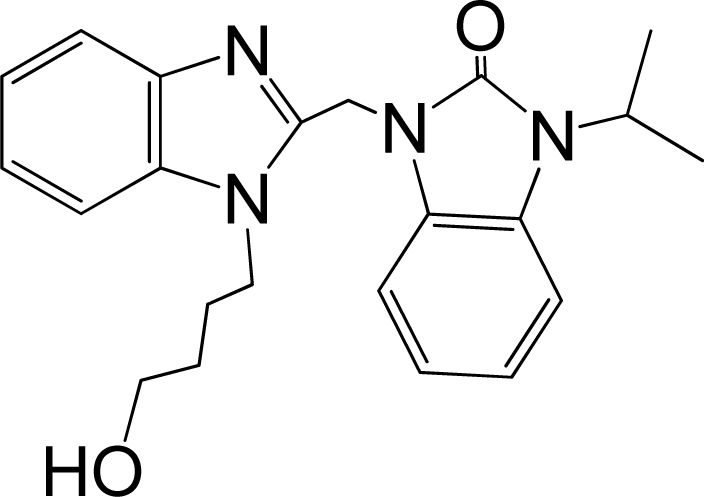	7.959	H	13
42^*^	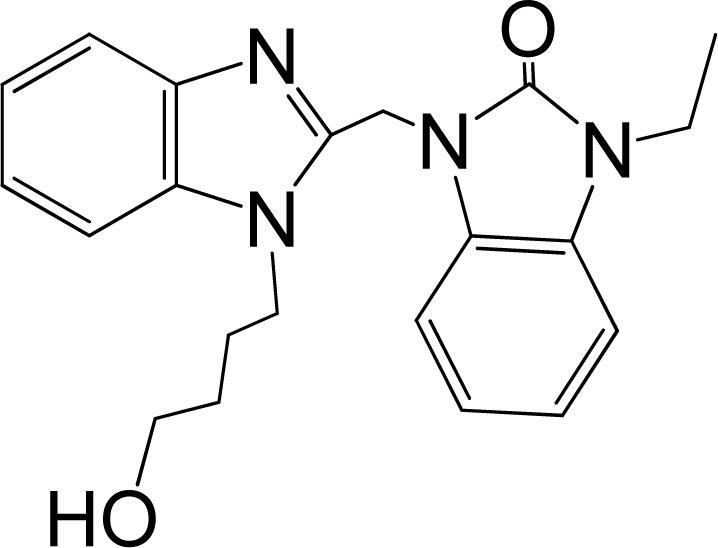	7.854	H	13
43	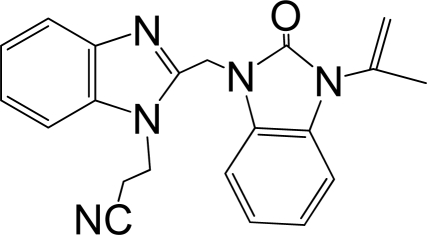	7.824	H	13

*, test set;

a, from the corresponding reference;

b, H denotes high active compounds, L denotes low active compounds.
