# Supplementary material for: A Classification Study of Respiratory Syncytial Virus (RSV) Inhibitors by Variable Selection with Random Forest
Source: Int J Mol Sci. 2011 Feb 21;12(2):1259–80. doi: 10.3390/ijms12021259 (PMC3083704; doi:10.3390/ijms12021259)
Supplement: Supplementary file 1 [file ijms-12-01259-s001.doc]

**Table S1.** Compounds with their chemical names, activities and classes used in the dataset.

| No. | Substituent | pEC_50_ | Class^b^ | Ref.^a^ |
| --- | --- | --- | --- | --- |
| 1 | H | 4.507 | L | 12 |
| 2 | CH_2_CH_2_NEt_2_ | 6.328 | L | 12 |
| 3 | CH_2_CH_2_N*i*Pr_2_ | 5.174 | L | 12 |
| 4^*^ | CH_2_CH_2_N(*c*CH_2_)_4_ | 6.222 | L | 12 |
| 5 | CH_2_CH_2_N(*c*CH_2_)_5_ | 5.959 | L | 12 |
| 6 | CH_2_CH_2_N(*c*CH_2_)_6_ | 5.553 | L | 12 |
| 7 | CH_2_CH_2_CH_2_NMe_2_ | 5.959 | L | 12 |
| 8^*^ | CHMe_2_ | 4.81 | L | 12 |
| 9 | CH_2_CH=CMe_2_ | 5.481 | L | 12 |
| 10 | CH_2_CH_2_OH | 5.114 | L | 12 |
| 11 | CH_2_CH_2_CN | 5.57 | L | 12 |
| 12^*^ | CH_2_CH_2_C(O)CH_3_ | 6.284 | L | 12 |
| 13^*^ | CH_2_CH_2_SOCH_3_ | 6.367 | L | 12 |
| 14 | CH_2_CH_2_CO_2_*t*Bu | 5.366 | L | 12 |
| 15 | CH_2_CH_2_CO_2_H | 4.742 | L | 12 |
| 16^*^ | CH_2_CH_2_CONH_2_ | 5.678 | L | 12 |
| 17 | CH_2_Ph | 4.979 | L | 12 |
| 18^*^ | CH_2_CH_2_Ph | 6.114 | L | 12 |
| 19^*^ | CH_2_C(O)Ph | 5.658 | L | 12 |
| 20 | CH_2_CH(OH)Ph | 6.229 | L | 12 |

| No. | Substituent | pEC_50_ | Class^b^ | Ref.^a^ |
| --- | --- | --- | --- | --- |
| 21 | CH_2_CH_2_N(*c*CH_2_)_4_ | 6.377 | L | 12 |
| 22 | CH_2_CH_2_N(*c*CH_2_)_5_ | 5.585 | L | 12 |
| 23 | CH_2_CH_2_N(*c*CH_2_)_6_ | 5.959 | L | 12 |
| 24 | CH_2_CH_2_CH_2_NMe_2_ | 5.444 | L | 12 |
| 25 | CH(CH_3_)_2_ | 4.288 | L | 12 |
| 26^*^ | CH_2_CH(CH_3_)_2_ | 4.323 | L | 12 |
| 27 | CH_2_CH_2_CH(CH_3_)_2_ | 6.357 | L | 12 |

| No. | Substituetnt | pEC_50_ | Class^b^ | Ref.^a^ |
| --- | --- | --- | --- | --- |
| 28 | CH_2_CH_2_NH_2_ | 5.481 | L | 12 |

**Table S1.** *Cont.*

| No. | R_1_ | R_2_ | pEC_50_ | Class^b^ | Ref.^a^ |
| --- | --- | --- | --- | --- | --- |
| 29 | H | *iso*-Propenyl | 6.125 | L | 13 |
| 30 | CH_2_CH_2_CH(CH_3_)_2_ | *iso*-Propenyl | 8.398 | H | 13 |
| 31 | CH_2_CH_2_CH(CH_3_)_2_ | CH_2_CH_3_ | 7.959 | H | 13 |
| 32^*^ | CH_2_CH_2_CH(CH_3_)_2_ | Allyl | 7.796 | H | 13 |
| 33^*^ | CH_2_CH_2_CH(CH_3_)_2_ | CH_2_CN | 7.553 | H | 13 |
| 34 | CH_2_CH_2_CH(CH_3_)_2_ | (CH_2_)_5_CN | 7.602 | H | 13 |
| 35 | CH_2_CH_2_CH(CH_3_)_2_ | CH_2_-4-C_6_H_4_-OH | 7.745 | H | 13 |
| 36 | CH_2_CH_2_CH(CH_3_)_2_ | CH_2_-4-C_6_H_4_-CN | 7.921 | H | 13 |
| 37 | CH_2_CH_2_CH(CH_3_)_2_ | CH_2_-4-C_6_H_4_-CON(CH_3_)_2_ | 7.678 | H | 13 |
| 38 | CH_2_CH_2_CH(CH_3_)_2_ | CH_2_-4-C_6_H_4_-SO_2_CH_3_ | 8.046 | H | 13 |
| 39^*^ | (CH_2_)_3_OH | Et | 8 | H | 13 |
| 40 | (CH_2_)_4_OH | *iso*-Propenyl | 8.398 | H | 13 |
| 41 | (CH_2_)_4_OH | *i*Pr | 7.959 | H | 13 |
| 42^*^ | (CH_2_)_4_OH | Et | 7.854 | H | 13 |
| 43 | (CH_2_)_2_CN | *iso*-Propenyl | 7.824 | H | 13 |
| 44 | (CH_2_)_2_CN | *i*Pr | 8.097 | H | 13 |
| 45 | (CH_2_)_2_CN | Ph | 8.301 | H | 13 |
| 46^*^ | (CH_2_)_4_CN | *iso*-Propenyl | 7.569 | H | 13 |
| 47^*^ | (CH_2_)_5_CN | *iso*-Propenyl | 7.886 | H | 13 |
| 48 | (CH_2_)_3_SO_2_CH_3_ | *iso*-Propenyl | 7.523 | H | 13 |
| 49 | (CH_2_)_3_SO_2_CH_3_ | H | 5.827 | L | 13 |
| 50 | CH_2_CH_2_CH(CH_3_)_2_ | SO_2_CH_3_ | 8.155 | H | 13 |
| 51^*^ |  | CH_2_-4-C_6_H_4_-OH | 8.398 | H | 13 |
| 52 | CH_2_CH_2_CH(CH_3_)_2_ | CH_2_-3,5-di-I-4-C_6_H_2_-OH | 6.164 | L | 13 |
| 53 | CH_2_CH_2_CH(CH_3_)_2_ | CH_2_-4-C_6_H_4_-SO_2_N(CH_3_)_2_ | 8.398 | H | 13 |
| 54 | (CH_2_)_6_CN | *iso*-Propenyl | 8.222 | H | 13 |
| 55 | (CH_2_)_6_CN | *i*Pr | 8.301 | H | 13 |
| 56^*^ | (CH_2_)_3_CN | (CH_2_)_4_CN | 7.602 | H | 13 |
| 57 | CH_2_CH_2_CH(CH_3_)_2_ | CH_2_CH_2_CO_2_H | 7.824 | H | 14 |
| 58 | CH_2_CH_2_CH(CH_3_)_2_ | (CH_2_)_4_CO_2_H | 8.097 | H | 14 |
| 59 | CH_2_CH_2_CH(CH_3_)_2_ | (CH_2_)_5_CO_2_H | 7.824 | H | 14 |
| 60 | CH_2_CH_2_CH(CH_3_)_2_ | CH_2_CONHSO_2_CH_3_ | 6.213 | L | 14 |
| 61 | CH_2_CH_2_CH(CH_3_)_2_ | CH_2_CONHSO_2_Ph | 5.916 | L | 14 |

**Table S1.** *Cont.*

| No. | R_1_ | R_2_ | pEC_50_ | Class^b^ | Ref.^a^ |
| --- | --- | --- | --- | --- | --- |
| 62 | CH_2_CH_2_CH(CH_3_)_2_ | CH_2_-4-C_6_H_4_-CO_2_H | 7.959 | H | 14 |
| 63 | CH_2_CH_2_CH(CH_3_)_2_ | CH_2_-3-C_6_H_4_-CO_2_H | 8 | H | 14 |
| 64 | CH_2_CH_2_CH(CH_3_)_2_ | CH_2_-2-C_6_H_4_-CO_2_H | 7.959 | H | 14 |
| 65 |  | *iso*-Propyl | 7.678 | H | 14 |
| 66 |  | CH_2_-4-C_6_H_4_-CO_2_H | 7.854 | H | 14 |
| 67^*^ |  | CH_2_-2-C_6_H_4_-SO_3_H | 7.886 | H | 14 |
| 68^*^ | (CH_2_)_3_OH | CH_2_CO_2_H | 5.714 | L | 14 |
| 69 | CH_2_CH_2_N(CH_3_)_2_ | *iso*-Proenyl | 7.959 | H | 14 |
| 70 | CH_2_CH_2_N(CH_3_)_2_ | *iso*-Propyl | 7.854 | H | 14 |
| 71^*^ | (CH_2_)_3_N(CH_3_)_2_ | H | 5.779 | L | 14 |
| 72^*^ | CH_2_CH_2_N(CH_3_)_2_ | CH_2_-4-C_6_H_4_-CO_2_CH_3_ | 7.638 | H | 14 |
| 73 | CH_2_CH_2_N(CH_3_)_2_ | CH_2_-2-C_6_H_4_-CO_2_CH_3_ | 7.721 | H | 14 |
| 74 | CH_2_CH_2_N(CH_3_)_2_ | CH_2_CH_2_N(CH_3_)_2_ | 6.398 | L | 14 |
| 75 | CH_2_CH_2_CH(CH_3_)_2_ | CH_2_-3,5-C_6_H_3_-(CO_2_H)_2_ | 6.183 | L | 14 |
| 76^*^ | CH_2_CH_2_CH(CH_3_)_2_ | CH_2_-4-C_6_H_4_-P(O)(OH)_2_ | 7.569 | H | 14 |
| 77 | CH_2_CH_2_CH(CH_3_)_2_ | CH_2_-2-C_6_H_4_-SO_3_H | 8.155 | H | 14 |
| 78 | CH_2_CH_2_CH(CH_3_)_2_ |  | 8.301 | H | 14 |
| 79 | CH_2_CH_2_CH(CH_3_)_2_ |  | 8.222 | H | 14 |
| 80 | CH_2_CH_2_CH(CH_3_)_2_ |  | 8.523 | H | 14 |
| 81 | CH_2_CH_2_CH(CH_3_)_2_ |  | 8.097 | H | 14 |
| 82^*^ |  | H | 5.922 | L | 14 |
| 83 |  | CH_2_-3-C_6_H_4_-CO_2_H | 8.155 | H | 14 |
| 84 |  | CH_2_-4-C_6_H_4_-P(O)(OH)_2_ | 6.434 | L | 14 |
| 85^*^ | (CH_2_)_4_OH | CH_2_-4-C_6_H_4_-CO_2_H | 8.398 | H | 14 |

**Table S1.** *Cont.*

| No. | R_2_ | pEC_50_ | Class^b^ | Ref.^a^ |
| --- | --- | --- | --- | --- |
| 86 | H | 5.243 | L | 15 |
| 87 | CH_2_-4-C_6_H_4_CN | 6.206 | L | 15 |

| No. | R_1_ | R_2_ | pEC_50_ | Class^b^ | Ref.^a^ |
| --- | --- | --- | --- | --- | --- |
| 88^*^ | (CH_2_)_3_CN | CH_2_-*c*Pr | 8.523 | H | 15 |
| 89^*^ | (CH_2_)_3_C(Me)_2_OH | *c*Pr | 7.87 | H | 15 |
| 90 | (CH_2_)_3_CF_3_ | *c*Pr | 8.222 | H | 15 |
| 91 | (CH_2_)_4_F | *c*Pr | 8 | H | 15 |
| 92 | (CH_2_)_4_F | CH_2_CF_3_ | 7.824 | H | 15 |

| No. | R_2_ | pEC_50_ | Class^b^ | Ref.^a^ |
| --- | --- | --- | --- | --- |
| 93^*^ |  | 8.523 | H | 15 |

| No. | R_1_ | R_2_ | pEC_50_ | Class^b^ | Ref.^a^ |
| --- | --- | --- | --- | --- | --- |
| 94 | (CH_2_)_3_CN | *i*Pr | 8.398 | H | 15 |
| 95 | (CH_2_)_3_CN | *t*Bu | 8.523 | H | 15 |
| 96 | (CH_2_)_3_CN | *c*Bu | 7.796 | H | 15 |
| 97 | (CH_2_)_3_CN | CH_2_CF_3_ | 7.824 | H | 15 |

**Table S1.** *Cont.*

| No. | R_1_ | R_2_ | pEC_50_ | Class^b^ | Ref.^a^ |
| --- | --- | --- | --- | --- | --- |
| 98 | (CH_2_)_5_OH | *c*Pr | 7.886 | H | 15 |
| 99^*^ | (CH_2_)_4_OH | *c*Bu | 8.155 | H | 15 |
| 100 | (CH_2_)_4_OH | *c*C_5_H_9_ | 8.097 | H | 15 |
| 101 | (CH_2_)_3_SO_2_CH_3_ | *c*Bu | 7.678 | H | 15 |
| 102 | (CH_2_)_3_SO_2_CH_3_ | CH_2_CF_3_ | 7.824 | H | 15 |
| 103 | (CH_2_)_3_SO_2_C_2_H_5_ | *c*Pr | 8.222 | H | 15 |
| 104^*^ | (CH_2_)_3_SO_2_C_2_H_5_ | CH_2_CF_3_ | 7.959 | H | 15 |
| 105^*^ | (CH_2_)_3_SO_2_*c*Pr | *c*Pr | 7.77 | H | 15 |
| 106 | (CH_2_)_3_CN | CHF_2_ | 7.959 | H | 15 |
| 107^*^ | (CH_2_)_3_SO_2_C_2_H_5_ | CHF_2_ | 7.959 | H | 15 |

| No. | R_1_ | R_2_ | pEC_50_ | Class^b^ | Ref.^a^ |
| --- | --- | --- | --- | --- | --- |
| 108 | 4-CH_2_NH_2_ | *i*Pr | 6.146 | L | 16 |
| 109 | 4-CH_2_OH | *i*Pr | 6.086 | L | 16 |
| 110 | 4-CH_2_OCH_3_ | *i*Pr | 5.125 | L | 16 |
| 111 | 4-CO_2_CH_3_ | *i*Pr | 3.638 | L | 16 |
| 112 | 4-CH_2_CN | *i*Pr | 3.618 | L | 16 |
| 113^*^ | 4-CH_2_CH_2_CN | *i*Pr | 3.631 | L | 16 |
| 114 | 4-CH_2_CH_2_CH_2_CN | *i*Pr | 3.672 | L | 16 |
| 115 | 5-Br | *i*Pr | 7.745 | H | 16 |
| 116 | 5-CN | *i*Pr | 6.322 | L | 16 |
| 117 | 5-CN | CH_2_CO_2_*t*Bu | 5.593 | L | 16 |
| 118 | 5-CH_2_=CH | *i*Pr | 6.201 | L | 16 |
| 119^*^ | 5-CH_3_CH_2_ | *i*Pr | 6.16 | L | 16 |
| 120^*^ |  | *i*Pr | 4.666 | L | 16 |
| 121 |  | Me | 3.622 | L | 16 |

**Table S1.** *Cont.*

| No. | R_1_ | R_2_ | pEC_50_ | Class^b^ | Ref.^a^ |
| --- | --- | --- | --- | --- | --- |
| 122 | 5-CO_2_H | Isopropenyl | 3.578 | L | 16 |
| 123 | 5-CO_2_H | *i*Pr | 3.623 | L | 16 |
| 124^*^ |  | *i*Pr | 3.648 | L | 16 |
| 125 | 5-CO_2_Me | *i*Pr | 3.638 | L | 16 |
| 126 | 5-C(O)N(CH_3_)_2_ | *i*Pr | 4.606 | L | 16 |
| 127 | 5-CH_2_NH_2_ | *i*Pr | 8.699 | H | 16 |
| 128 | 5-CH_2_CH_2_NH_2_ | *i*Pr | 5.364 | L | 16 |
| 129 | 5-CH_2_N(CH_3_)_2_ | *i*Pr | 3.738 | L | 16 |
| 130 | 5-CH_2_NHC(O)CH_3_ | *i*Pr | 6.2 | L | 16 |
| 131^*^ | 5-CH_2_NHS(O)_2_Me | *i*Pr | 6.14 | L | 16 |
| 132 | 5-C(CH_3_)NH_2_ | *i*Pr | 6.159 | L | 16 |
| 133 | 5-C(NH)NH_2_ | Isopropenyl | 8.398 | H | 16 |
| 134 | 5-C(NH)NH_2_ | CH_2_CO_2_*t*Bu | 8.046 | H | 16 |
| 135 | 5-C(NH)NH_2_ | CH_2_CO_2_H | 7.509 | H | 16 |
| 136 | 5-C(NOH)NH_2_ | Isopropenyl | 8.301 | H | 16 |
| 137 | 5-C(NOH)NH_2_ | CH_2_CO_2_*t*Bu | 7.796 | H | 16 |
| 138 | 6-CH_2_NHCH_3_ | *i*Pr | 3.728 | L | 16 |
| 139 | 6-CH_2_N(CH_3_)_2_ | *i*Pr | 3.74 | L | 16 |
| 140^*^ | 6-CH_2_CH_2_NH_2_ | *i*Pr | 5.148 | L | 16 |
| 141 | 6-CO_2_H | *i*Pr | 3.623 | L | 16 |
| 142^*^ | 6-C(O)NH_2_ | *i*Pr | 3.91 | L | 16 |
| 143 | 7-CH_2_NH_2_ | *i*Pr | 3.717 | L | 16 |
| 144^*^ | 7-CO_2_CH_3_ | *i*Pr | 3.638 | L | 16 |
| 145^*^ | 7-CH_2_OH | *i*Pr | 3.609 | L | 16 |

| No. | R | R_1_ | X | Y | pEC_50_ | Class^b^ | Ref.^a^ |
| --- | --- | --- | --- | --- | --- | --- | --- |
| 146 | H | (CH_2_)_2_CH(CH_3_)_2_ | CHCH_2_CO_2_H | - | 6.152 | L | 17 |
| 147 | H | (CH_2_)_2_CH(CH_3_)_2_ | CHCH_2_CONHCH_2_CH_2_Ph | - | 6.421 | L | 17 |
| 148^*^ | H | (CH_2_)_4_F | CH | CH | 5.637 | L | 17 |

**Table S1.** *Cont.*

| No. | R | R_1_ | X | Y | pEC_50_ | Class^b^ | Ref.^a^ |
| --- | --- | --- | --- | --- | --- | --- | --- |
| 149 | H | (CH_2_)_4_F | CEt | CH | 6.045 | L | 17 |
| 150 | NH_2_CH_2_ | (CH_2_)_4_F | CH | C(OCH_3_) | 8.046 | H | 17 |
| 151 | NH_2_CH_2_ | (CH_2_)_4_OH | CH | C(OCH_3_) | 8.097 | H | 17 |
| 152 | H | (CH_2_)_4_F | C(Br) | CH | 6.103 | L | 17 |
| 153 | H | (CH_2_)_4_CN | CH_2_ | CH_2_ | 5.956 | L | 17 |
| 154^*^ | H | (CH_2_)_2_N(CH_3_)_2_ | CH_2_ | CH_2_ | 6.341 | L | 17 |
| 155^*^ | NH_2_CH_2_ | (CH_2_)_4_F | NCH_2_CF_3_ | C=O | 8.097 | H | 17 |
| 156 | NH_2_CH_2_ | (CH_2_)_4_OH | NCH_2_CF_3_ | C=O | 8.097 | H | 17 |
| 157 | H | (CH_2_)_2_CH(CH_3_)_2_ | NCH_2_CO_2_Et | C=O | 5.732 | L | 17 |
| 158 | H | (CH_2_)_2_CH(CH_3_)_2_ | NCH_2_-4-C_6_H_4_CO_2_H | C=O | 8.046 | H | 17 |
| 159 | H | (CH_2_)_2_N(CH_3_)_2_ | NCH_2_-4-C_6_H_4_CO_2_Me | C=O | 6.379 | L | 17 |
| 160 | H | (CH_2_)_4_F | C(CH_3_)_2_ | SO_2_ | 5.115 | L | 17 |
| 161 | H | (CH_2_)_4_OH | NCH_3_ | SO_2_ | 7.721 | H | 17 |
| 162 | H | (CH_2_)_3_SO_2_CH_3_ | NCH_3_ | SO_2_ | 5.242 | L | 17 |
| 163 | H | (CH_2_)_3_CN | O | - | 5.6 | L | 17 |
| 164^*^ | H | (CH_2_)_3_CN | C(CH_3_)_2_ | - | 7.721 | H | 17 |
| 165^*^ | H | (CH_2_)_4_F | C(OH) | C(CO_2_H) | 4.586 | L | 17 |
| 166 | H | (CH_2_)_4_F | C(OH) | CH | <3.563 | L | 17 |
| 167 | NH_2_CH_2_ | (CH_2_)_3_SO_2_CH_3_ | NCH_2_CF_3_ | C=O | 7.678 | H | 17 |
| 168 | NH_2_CH_2_ | (CH_2_)_3_SO_2_CH_3_ | NCH_2_CO_2_H | C=O | 5.993 | L | 17 |
| 169^*^ | H | (CH_2_)_4_CN | CH_2_ | SO_2_ | 5.873 | L | 17 |
| 170 | H | (CH_2_)_4_F | CH_2_ | S | 5.991 | L | 17 |
| 171 | H | (CH_2_)_4_F | C(CH_3_)_2_ | S | <3.599 | L | 17 |
| 172 | NH_2_CH_2_ | (CH_2_)_2_CH(CH_3_)_2_ | N*c*C_3_H_5_ | C=O | 7.959 | H | 17 |
| 173^*^ | CH_3_NHCH_2_ | (CH_2_)_2_CH(CH_3_)_2_ | N*c*C_3_H_5_ | C=O | 6.103 | L | 17 |
| 174 | *n*C_4_H_9_NHCH_2_ | (CH_2_)_2_CH(CH_3_)_2_ | N*c*C_3_H_5_ | C=O | <3.719 | L | 17 |
| 175 | CO_2_CH_3_ | (CH_2_)_2_CH(CH_3_)_2_ | N*c*C_3_H_5_ | C=O | <3.663 | L | 17 |
| 176 | CO_2_H | (CH_2_)_2_CH(CH_3_)_2_ | N*c*C_3_H_5_ | C=O | <4.163 | L | 17 |
| 177 | NH_2_CH_2_ | (CH_2_)_4_F | N*c*C_3_H_5_ | C=O | 8.046 | H | 17 |
| 178 | H | (CH_2_)_4_OH | N*c*C_3_H_5_ | C=O | 7.602 | H | 17 |
| 179^*^ | NH_2_CH_2_ | (CH_2_)_4_OH | N*c*C_3_H_5_ | C=O | 7.745 | H | 17 |
| 180 | H | (CH_2_)_4_F | N*c*C_3_H_5_ | SO_2_ | 6.365 | L | 17 |
| 181 | NH_2_CH_2_ | (CH_2_)_2_CH(CH_3_)_2_ | N*c*C_3_H_5_ | SO_2_ | 8.523 | H | 17 |

**Table S1.** *Cont.*

| No. | Side chain (SC) | R_1_ | pEC_50_ | Class^b^ | Ref.^a^ |
| --- | --- | --- | --- | --- | --- |
| 182 | -(CH_2_)_2_CH(Me)_2_ | H | 8 | H | 18 |
| 183 | -(CH_2_)_4_OH | Me | 8 | H | 18 |
| 184^*^ | -(CH_2_)_4_OAc | Me | 7.523 | H | 18 |
| 185 | -(CH_2_)_3_OMe | Me | 7.523 | H | 18 |
| 186 | -(CH_2_)_3_OMe | -CH_2_CH_3_ | 5.562 | L | 18 |
| 187 | -(CH_2_)_4_OH | *t*Bu | 5.947 | L | 18 |
| 188 | -(CH_2_)_3_OMe | -CH(CH_2_CH_3_)_2_ | 6.029 | L | 18 |
| 189^*^ | -(CH_2_)_3_OMe | -Cyclo-hexyl | 6.186 | L | 18 |
| 190 | -(CH_2_)_3_OMe | -(CH_2_)_2_*c*Hex | 6.416 | L | 18 |
| 191 | -(CH_2_)_3_OMe | -CH_2_CH=CH_2_ | 6.369 | L | 18 |
| 192^*^ | -(CH_2_)_3_OMe | -CH_2_CONEt_2_ | 7.585 | H | 18 |
| 193 | -(CH_2_)_3_OMe | -(CH_2_)_2_NMe_2_ | 6.432 | L | 18 |
| 194 | -(CH_2_)_3_OMe | -(CH_2_)_2_piperidine | 6.166 | L | 18 |
| 195 | -(CH_2_)_3_OMe | -CH(CO_2_H)CH_2_CO_2_H | 7.721 | H | 18 |
| 196 | -(CH_2_)_2_CHMe_2_ | Ph | 6.426 | L | 18 |
| 197 | -(CH_2_)_2_CHMe_2_ | 4’-Ph-Br | 6.152 | L | 18 |
| 198 | -(CH_2_)_2_CHMe_2_ | -CH_2_-Ph-4-CO_2_H | 7.796 | H | 18 |
| 199 | -(CH_2_)_2_NMe_2_ | -CH_2_-4’-Ph-CO_2_H | 7.783 | H | 18 |
| 200 | -(CH_2_)_2_CHMe_2_ | -CH_2_-4’-Ph-CO_2_Me | 7.638 | H | 18 |
| 201 | -(CH_2_)_2_NMe_2_ | -CH_2_-4’-Ph-CO_2_Me | 7.638 | H | 18 |
| 202^*^ | -(CH_2_)_3_OMe | -CH_2_-4’-Ph-CONMe_2_ | 7.523 | H | 18 |
| 203 | -(CH_2_)_3_CHMe_2_ | -CH_2_-4’-Ph-CONMe_2_ | 8.046 | H | 18 |
| 204 | -(CH_2_)_4_OH | -CH_2_-4’-Ph-SO_2_Me | 7.921 | H | 18 |
| 205 | -(CH_2_)OAc | -CH_2_-4’-Ph-SO_2_Me | 8.301 | H | 18 |

| No. | Aza position | SC | R_1_ | pEC_50_ | Class^b^ | Ref.^a^ |
| --- | --- | --- | --- | --- | --- | --- |
| 206 | 6 | -(CH_2_)_4_-F | -H | 7.747 | H | 18 |
| 207 | 7 | -(CH_2_)_3_-CN | -H | 7.644 | H | 18 |
| 208 | 6 | -(CH_2_)_4_OH | -CH_3_ | 7.51 | H | 18 |
| 209^*^ | 7 | -(CH_2_)_4_OH | -CH_3_ | 7.506 | H | 18 |
| 210 | 6 | -(CH_2_)_3_SO_2_Me | -CH_2_CH_2_F | 7.903 | H | 18 |

**Table S1.** *Cont.*

| No. | Aza position | SC | R_1_ | pEC_50_ | Class^b^ | Ref.^a^ |
| --- | --- | --- | --- | --- | --- | --- |
| 211^*^ | 7 | -(CH_2_)_3_-SO_2_Me | -CH_2_CH_2_F | 7.535 | H | 18 |
| 212 | 6 | -(CH_2_)_3_SO_2_Me | -CH_2_CH=CH_2_ | 7.907 | H | 18 |
| 213 | 6 | -(CH_2_)_3_SO_2_Me | -CH_2_-2-pyridine | 7.538 | H | 18 |
| 214 | 7 | -(CH_2_)_4_OH | -CH_2_-4’-Ph-SO_2_CH_3_ | 8.137 | H | 18 |
| 215 | 5 | -(CH_2_)_3_SO_2_Me | -CH_2_-4’-Ph-SO_2_CH_3_ | 4.975 | L | 18 |
| 216 | 5 | -(CH_2_)_3_CN | -CH_2_-4’-Ph-SO_2_CH_3_ | 4.992 | L | 18 |

^*^, test set; ^a^, from the corresponding reference;

^b^, H denotes high active compounds, L denotes low active compounds.
