# Supplementary material for: A Classification Study of Respiratory Syncytial Virus (RSV) Inhibitors by Variable Selection with Random Forest
Source: Int J Mol Sci. 2011 Feb 21;12(2):1259–80. doi: 10.3390/ijms12021259 (PMC3083704; doi:10.3390/ijms12021259)
Supplement: Supplementary file 2 [file ijms-12-01259-s003.doc]

**Table S3.** Compounds with their observed and predicted classes using the VS-RF, SVM, GP, LDA, and *k*NN models, respectively, in the dataset.

| No. | pEC_50_ | Obs. Class^a^ | Pred. class^a^ | | | | |
| --- | --- | --- | --- | --- | --- | --- | --- |
|  |  |  | VS-RF | SVM | GP | LDA | *k*NN |
| 1 | 4.507 | L | L | L | L | L | L |
| 2 | 6.328 | L | L | L | L | L | L |
| 3 | 5.174 | L | L | L | L | H | H |
| 4^*^ | 6.222 | L | L | L | L | L | L |
| 5 | 5.959 | L | L | L | L | L | L |
| 6 | 5.553 | L | L | L | L | L | L |
| 7 | 5.959 | L | L | L | L | L | L |
| 8^*^ | 4.81 | L | L | L | L | L | L |
| 9 | 5.481 | L | L | L | L | L | L |
| 10 | 5.114 | L | L | L | L | L | L |
| 11 | 5.57 | L | L | L | L | L | L |
| 12^*^ | 6.284 | L | L | L | L | L | L |
| 13^*^ | 6.367 | L | L | L | L | L | L |
| 14 | 5.366 | L | L | L | L | L | L |
| 15 | 4.742 | L | L | L | L | L | L |
| 16^*^ | 5.678 | L | L | L | L | L | L |
| 17 | 4.979 | L | L | L | L | L | L |
| 18^*^ | 6.114 | L | L | L | L | L | L |
| 19^*^ | 5.658 | L | L | L | L | L | H |
| 20 | 6.229 | L | L | L | L | L | H |
| 21 | 6.377 | L | L | L | L | L | L |
| 22 | 5.585 | L | L | L | L | L | L |
| 23 | 5.959 | L | L | L | L | L | L |
| 24 | 5.444 | L | L | L | L | L | L |
| 25 | 4.288 | L | L | L | L | L | L |
| 26^*^ | 4.323 | L | L | L | L | L | L |
| 27 | 6.357 | L | L | L | L | L | L |
| 28 | 5.481 | L | L | L | L | L | L |
| 29 | 6.125 | L | L | L | H | H | L |
| 30 | 8.398 | H | H | H | H | L | H |
| 31 | 7.959 | H | H | H | H | L | H |
| 32^*^ | 7.796 | H | H | H | H | L | H |
| 33^*^ | 7.553 | H | H | H | H | H | H |
| 34 | 7.602 | H | H | H | H | L | H |
| 35 | 7.745 | H | H | H | H | L | H |
| 36 | 7.921 | H | H | H | H | L | H |
| 37 | 7.678 | H | H | H | H | H | H |
| 38 | 8.046 | H | H | H | H | H | H |
| 39^*^ | 8 | H | H | H | H | H | H |

***Table S3.*** *Cont.*

| No. | pEC_50_ | Obs. class^a^ | Pred. class^a^ | | | | |
| --- | --- | --- | --- | --- | --- | --- | --- |
|  |  |  | VS-RF | SVM | GP | LDA | *k*NN |
| 40 | 8.398 | H | H | H | H | L | H |
| 41 | 7.959 | H | H | H | H | L | H |
| 42^*^ | 7.854 | H | H | H | H | H | H |
| 43 | 7.824 | H | H | H | H | H | H |
| 44 | 8.097 | H | H | H | H | H | H |
| 45 | 8.301 | H | H | H | H | H | H |
| 46^*^ | 7.569 | H | H | H | H | H | H |
| 47^*^ | 7.886 | H | H | H | H | H | H |
| 48 | 7.523 | H | H | H | H | H | H |
| 49 | 5.827 | L | L | L | L | H | L |
| 50 | 8.155 | H | H | H | H | H | H |
| 51^*^ | 8.398 | H | H | H | H | L | H |
| 52 | 6.164 | L | L | L | H | H | H |
| 53 | 8.398 | H | H | H | H | H | H |
| 54 | 8.222 | H | H | H | H | L | H |
| 55 | 8.301 | H | H | H | H | L | H |
| 56^*^ | 7.602 | H | H | H | H | H | H |
| 57 | 7.824 | H | H | H | H | L | H |
| 58 | 8.097 | H | H | H | H | L | H |
| 59 | 7.824 | H | H | H | H | L | H |
| 60 | 6.213 | L | L | L | H | H | L |
| 61 | 5.916 | L | L | L | L | H | H |
| 62 | 7.959 | H | H | H | H | L | H |
| 63 | 8 | H | H | H | H | L | H |
| 64 | 7.959 | H | H | H | H | H | H |
| 65 | 7.678 | H | H | H | H | H | L |
| 66 | 7.854 | H | H | H | H | H | H |
| 67^*^ | 7.886 | H | H | L | H | H | H |
| 68^*^ | 5.714 | L | H | H | H | H | H |
| 69 | 7.959 | H | H | H | H | H | H |
| 70 | 7.854 | H | H | H | H | L | H |
| 71 | 5.779 | L | L | L | L | H | L |
| 72 | 7.638 | H | H | H | H | H | H |
| 73 | 7.721 | H | H | H | H | H | H |
| 74 | 6.398 | L | L | L | H | H | H |
| 75 | 6.183 | L | L | L | H | H | H |
| 76^*^ | 7.569 | H | H | L | H | L | H |
| 77 | 8.155 | H | H | H | H | H | H |
| 78 | 8.301 | H | H | H | H | H | H |

***Table S3.*** *Cont.*

| No. | pEC_50_ | Obs. class^a^ | Pred. class^a^ | | | | |
| --- | --- | --- | --- | --- | --- | --- | --- |
|  |  |  | VS-RF | SVM | GP | LDA | *k*NN |
| 79 | 8.222 | H | H | H | H | H | H |
| 80 | 8.523 | H | H | H | H | H | H |
| 81 | 8.097 | H | H | H | H | H | H |
| 82^*^ | 5.922 | L | L | L | L | L | H |
| 83 | 8.155 | H | H | H | H | H | H |
| 84 | 6.434 | L | L | L | H | H | H |
| 85^*^ | 8.398 | H | H | H | H | H | H |
| 86 | 5.243 | L | L | L | L | H | L |
| 87 | 6.206 | L | L | H | H | H | H |
| 88^*^ | 8.523 | H | H | H | H | H | H |
| 89^*^ | 7.87 | H | H | H | H | H | L |
| 90 | 8.222 | H | H | H | H | H | L |
| 91 | 8 | H | H | H | H | H | H |
| 92 | 7.824 | H | H | H | H | H | H |
| 93^*^ | 8.523 | H | H | H | H | H | H |
| 94 | 8.398 | H | H | H | H | H | H |
| 95 | 8.523 | H | H | H | H | H | H |
| 96 | 7.796 | H | H | H | H | H | L |
| 97 | 7.824 | H | H | H | H | H | H |
| 98 | 7.886 | H | H | H | H | H | H |
| 99^*^ | 8.155 | H | H | H | H | H | L |
| 100 | 8.097 | H | H | H | H | H | H |
| 101 | 7.678 | H | H | H | H | H | H |
| 102 | 7.824 | H | H | H | H | H | L |
| 103 | 8.222 | H | H | H | H | H | L |
| 104^*^ | 7.959 | H | H | H | H | H | L |
| 105^*^ | 7.77 | H | H | H | H | H | L |
| 106 | 7.959 | H | H | H | H | H | H |
| 107^*^ | 7.959 | H | H | H | H | H | H |
| 108 | 6.146 | L | L | L | L | L | L |
| 109 | 6.086 | L | L | L | L | L | L |
| 110 | 5.125 | L | L | L | L | L | L |
| 111 | 3.638 | L | L | L | L | L | H |
| 112 | 3.618 | L | L | L | L | L | L |
| 113^*^ | 3.631 | L | L | L | L | L | L |
| 114 | 3.672 | L | L | L | L | L | L |
| 115 | 7.745 | H | H | L | L | L | H |
| 116 | 6.322 | L | L | L | L | L | L |
| 117 | 5.593 | L | L | L | H | H | H |

**Table S3.** *Cont.*

| No. | pEC_50_ | Obs. class^a^ | Pred. class^a^ | | | | |
| --- | --- | --- | --- | --- | --- | --- | --- |
|  |  |  | VS-RF | SVM | GP | LDA | *k*NN |
| 118 | 6.201 | L | L | L | L | L | L |
| 119^*^ | 6.16 | L | L | L | L | L | L |
| 120^*^ | 4.666 | L | H | H | H | H | H |
| 121 | 3.622 | L | L | H | H | H | H |
| 122 | 3.578 | L | L | L | L | H | L |
| 123 | 3.623 | L | L | L | L | L | L |
| 124^*^ | 3.648 | L | H | H | H | H | H |
| 125 | 3.638 | L | L | L | L | H | H |
| 126 | 4.606 | L | L | L | L | H | H |
| 127 | 8.699 | H | H | L | L | L | L |
| 128 | 5.364 | L | L | L | L | L | L |
| 129 | 3.738 | L | L | L | L | H | L |
| 130 | 6.2 | L | L | L | L | L | L |
| 131^*^ | 6.14 | L | L | H | L | H | H |
| 132 | 6.159 | L | L | L | L | L | H |
| 133 | 8.398 | H | H | L | L | H | L |
| 134 | 8.046 | H | H | H | H | H | H |
| 135 | 7.509 | H | H | H | H | H | H |
| 136 | 8.301 | H | H | L | L | H | H |
| 137 | 7.796 | H | H | H | H | H | H |
| 138 | 3.728 | L | L | L | L | L | L |
| 139 | 3.74 | L | L | L | L | H | L |
| 140^*^ | 5.148 | L | L | L | L | L | L |
| 141 | 3.623 | L | L | L | L | L | L |
| 142^*^ | 3.91 | L | L | L | L | L | L |
| 143 | 3.717 | L | L | L | L | L | L |
| 144^*^ | 3.638 | L | L | L | L | L | L |
| 145^*^ | 3.609 | L | L | L | L | L | L |
| 146 | 6.152 | L | L | L | H | L | H |
| 147 | 6.421 | L | L | L | H | L | L |
| 148^*^ | 5.637 | L | L | L | L | L | L |
| 149 | 6.045 | L | L | L | L | L | H |
| 150 | 8.046 | H | H | H | L | L | H |
| 151 | 8.097 | H | H | H | L | L | H |
| 152 | 6.103 | L | L | L | L | L | L |
| 153 | 5.956 | L | L | L | L | L | L |
| 154^*^ | 6.341 | L | L | L | L | L | L |
| 155^*^ | 8.097 | H | H | H | H | L | H |
| 156 | 8.097 | H | H | H | H | L | H |

***Table S3.*** *Cont.*

| No. | pEC_50_ | Obs. class^a^ | Pred. class^a^ | | | | |
| --- | --- | --- | --- | --- | --- | --- | --- |
|  |  |  | VS-RF | SVM | GP | LDA | *k*NN |
| 157 | 5.732 | L | L | L | L | L | L |
| 158 | 8.046 | H | H | H | L | L | H |
| 159 | 6.379 | L | L | L | L | L | H |
| 160 | 5.115 | L | L | L | L | L | H |
| 161 | 7.721 | H | H | H | H | H | H |
| 162 | 5.242 | L | L | L | H | H | H |
| 163 | 5.6 | L | L | L | L | L | L |
| 164^*^ | 7.721 | H | H | L | H | L | H |
| 165^*^ | 4.586 | L | L | L | H | L | H |
| 166 | 3.563 | L | L | L | L | L | L |
| 167 | 7.678 | H | H | H | H | H | H |
| 168 | 5.993 | L | L | L | H | L | L |
| 169^*^ | 5.873 | L | L | H | H | L | H |
| 170 | 5.991 | L | L | L | L | L | L |
| 171 | 3.599 | L | L | L | L | L | H |
| 172 | 7.959 | H | H | L | L | L | H |
| 173^*^ | 6.103 | L | L | L | L | L | H |
| 174 | 3.719 | L | L | L | L | L | H |
| 175 | 3.663 | L | L | L | L | H | H |
| 176 | 4.163 | L | L | L | L | H | H |
| 177 | 8.046 | H | H | H | H | H | H |
| 178 | 7.602 | H | H | H | H | L | L |
| 179^*^ | 7.745 | H | H | H | H | L | H |
| 180 | 6.365 | L | L | L | L | H | H |
| 181 | 8.523 | H | H | H | H | H | H |
| 182 | 8 | H | H | H | H | L | H |
| 183 | 8 | H | H | H | H | L | H |
| 184^*^ | 7.523 | H | H | H | H | L | H |
| 185 | 7.523 | H | H | H | H | L | H |
| 186 | 5.562 | L | L | L | H | H | L |
| 187 | 5.947 | L | L | L | L | L | L |
| 188 | 6.029 | L | L | L | H | L | L |
| 189^*^ | 6.186 | L | L | L | H | L | L |
| 190 | 6.416 | L | L | L | L | L | L |
| 191 | 6.369 | L | L | L | H | L | H |
| 192^*^ | 7.585 | H | H | L | H | H | L |
| 193 | 6.432 | L | L | L | L | L | H |
| 194 | 6.166 | L | L | L | H | L | L |
| 195 | 7.721 | H | H | L | H | L | L |

***Table S3.*** *Cont.*

| No. | pEC_50_ | Obs. class^a^ | Pred. class^a^ | | | | |
| --- | --- | --- | --- | --- | --- | --- | --- |
|  |  |  | VS-RF | SVM | GP | LDA | *k*NN |
| 196 | 6.426 | L | L | L | L | L | L |
| 197 | 6.152 | L | L | L | L | L | L |
| 198 | 7.796 | H | H | H | H | L | H |
| 199 | 7.783 | H | H | H | H | H | H |
| 200 | 7.638 | H | H | H | H | H | H |
| 201 | 7.638 | H | H | H | H | H | H |
| 202^*^ | 7.523 | H | H | H | H | H | H |
| 203 | 8.046 | H | H | H | H | H | H |
| 204 | 7.921 | H | H | H | H | H | H |
| 205 | 8.301 | H | H | H | H | H | H |
| 206 | 7.747 | H | H | H | H | H | H |
| 207 | 7.644 | H | H | H | H | H | H |
| 208 | 7.51 | H | H | H | H | H | H |
| 209^*^ | 7.506 | H | H | H | H | H | H |
| 210 | 7.903 | H | H | H | H | H | H |
| 211^*^ | 7.535 | H | H | H | H | H | H |
| 212 | 7.907 | H | H | H | H | H | H |
| 213 | 7.538 | H | H | H | H | H | L |
| 214 | 8.137 | H | H | H | H | H | H |
| 215 | 4.975 | L | L | L | H | H | H |
| 216 | 4.992 | L | L | H | H | H | H |

^*^, test set; ^a^, H denotes high active compounds, L denotes low active compounds.
